# Autophagy-Mediated Inflammatory Cytokine Secretion in Sporadic ALS Patient iPSC-Derived Astrocytes

**DOI:** 10.1155/2022/6483582

**Published:** 2022-08-22

**Authors:** BaofengFeng, Asiamah Ernest Amponsah, Ruiyun Guo, Xin Liu, Jinyu Zhang, Xiaofeng Du, Zijing Zhou, Jingjing He, Jun Ma, Huixian Cui

**Affiliations:** ^1^Hebei Medical University-National University of Ireland Galway Stem Cell Research Center, Hebei Medical University, Hebei Province 050017, China; ^2^Hebei Research Center for Stem Cell Medical Translational Engineering, Hebei Province 050017, China; ^3^Human Anatomy Department, Hebei Medical University, Hebei Province 050017, China

## Abstract

**Background:**

Astrocytes can be involved in motor neuron toxicity in amyotrophic lateral sclerosis (ALS) induced by noncell autonomous effects, and inflammatory cytokines may play the main role in mediating this process. However, the etiology of aberrant cytokine secretion is unclear. The present study assessed possible involvement of the mTOR-autophagy pathway in aberrant cytokine secretion by ALS patient iPSC-derived astrocytes. *Method and Results*. PBMCs from sporadic ALS patients and control subjects were reprogrammed into iPSCs, which were then differentiated into astrocytes and/or motor neurons. Comparison with control astrocytes indicated that conditioned medium of ALS astrocytes reduced the viability of the control motor neurons (*p* < 0.05) assessed using the MTT assay. The results of ELISA showed that the concentrations of TNF*α*, IL1*β*, and IL6 in cell culture medium of ALS astrocytes were increased (*p* < 0.05). ALS astrocytes had higher p62 and mTOR levels and lower LC3BII/LC3BI ratio and ULK1 and p-Beclin-1 (Ser15) levels (*p* < 0.05), indicating defective autophagy. Exogenous inhibition of the mTOR-autophagy pathway, but not the activation of the pathway in control subject astrocytes, increased the levels of p62 and mTOR and concentration of IL-1*β*, TNF-*α*, and IL-6 in cell culture medium and decreased the LC3BII/LC3BI ratio and levels of ULK1 and p-Beclin-1 (Ser15), and these changes were comparable to those in ALS astrocytes. After 48 h of rapamycin (autophagy activator) and 3-methyladenine (autophagy inhibitor) treatments, the exogenous activation of the mTOR-autophagy pathway, but not inhibition of the pathway, in ALS astrocytes significantly reduced the concentrations of TNF*α*, IL1*β*, and IL6 in cell culture medium and reduced the levels of p62, while increasing the levels of LC3B-II/LC3B-I, ULK1, and p-Beclin-1 (Ser15), and these changes were comparable to those in control subject astrocytes.

**Conclusion:**

Alteration in the mTOR/ULK1/Beclin-1 pathway regulated cytokine secretion in ALS astrocytes, which was able to lead to noncell autonomous toxicity. Autophagy activation mitigated cytokine secretion by ALS astrocytes.

## 1. Introduction

Amyotrophic lateral sclerosis (ALS) is a progressive neurodegenerative disease that affects upper and lower motor neurons [1]. ALS leads to motor neuron-related pathologies, extra motor deficits, and even death [2]. Based on genetics, ALS is classified as familial ALS, which is associated with mutations in the ALS-linked genes, and sporadic ALS, which has a multifactorial etiology. Recent reports have shown that noncell autonomous mechanisms involving non-neuronal cells, such as astrocytes, mediate motor neuron toxicity. Sporadic and familial ALS astrocytes derived from postmortem brain [3], directly converted fibroblasts [4], iPSC-derived astrocytes [5], and/or embryonic stem cells [6] exhibit toxicity against the motor neurons in vitro and in vivo [5]. However, most of the studies that investigated the mechanisms underlying astrocyte-mediated motor neuron toxicity focused on familial ALS [7–10], which accounts for up to 10% of all ALS cases. The data on sporadic ALS astrocyte-mediated motor neuron toxicity are rather scarce.

Inflammation is recognized as one of the causes of noncell autonomous toxicity in ALS pathogenesis. Sporadic and familial ALS brain, spinal cord, sera, and cerebrospinal fluid (CSF) exhibit upregulation of inflammatory cytokines, such as tumor necrosis factor-alpha (TNF-*α*), interleukin 6 (IL6), and interleukin 1*β* (IL1*β*) [11–14]. In the CNS, astrocytes are one of the major inducible sources of the cytokines [15, 16]. Cytokine-mediated motor neuron toxicity has been demonstrated in familial ALS in the case of FUS-mutated astrocytes that secrete high levels of TNF*α* to cause MN toxicity [9]. However, it is not known whether sporadic ALS astrocytes exhibit aberrant cytokine secretion in vitro. Moreover, the etiology of aberrant cytokine secretion in ALS astrocytes is unknown.

Autophagy has been proposed to regulate inflammation [17]. Autophagy is a tightly regulated intracellular catabolic process dedicated to lysosome-mediated degradation of the cytoplasmic materials, such as protein aggregates and defunct organelles [17]. Autophagy and inflammation reciprocally regulate each other. On the one hand, autophagy inhibits or clears proteins and defunct organelles that can induce an inflammatory response and influences the development, homeostasis and survival of inflammatory cells and transcription, processing, and secretion of the cytokines [17–19]. On the other hand, inflammatory cytokines interact with the plasma membrane-bound receptors to activate or inhibit the downstream autophagy-relevant signaling pathways [20]. Similar to other neurodegenerative diseases, autophagy dysfunction is involved in ALS pathogenesis [21]; specifically, ALS is characterized by the accumulation of cytoplasmic inclusions and misfolded proteins, such as sequestosome (p62) and TARDBP/TDP-43 (TAR DNA-binding protein) in the motor neurons and/or neuroglia [22–24]. Defective autophagy has been implicated in motor neuron toxicity. Familial ALS astrocyte hSOD1 impaired autophagy in the motor neurons to cause motor neuron toxicity. A recent report indicated that autophagy is dysregulated not only in the motor neurons but also in astrocytes, such as ALS SOD1^G93A^ astrocytes. Autophagy is impaired in ALS SOD1^G93A^ astrocytes, and the modulation of the IGF1R-mTOR (insulin-like growth factor 1 receptor-mammalian target of rapamycin) pathway attenuates motor neuron toxicity in astrocytes [8]. Based on this finding, autophagy dysregulation may be able to mediate aberrant cytokine secretion.

Therefore, the present study aimed to investigate whether sporadic ALS astrocytes exhibit aberrant cytokine secretion and a dysfunctional autophagy pathway. The present study further assessed the potential of dysfunctional autophagy to cause aberrant cytokine secretion from the viewpoint of autophagy-regulating cytokine secretion, leading to astrocyte toxicity in sporadic ALS. We reprogrammed peripheral blood mononuclear cells (PBMCs) from ALS patients and healthy control subjects into iPSCs (induced pluripotent stem cells), which were eventually differentiated into mature astrocytes. First, the motor neuron-supportive potential and cytokine (IL1*β*, TNF*α*, and IL6) concentrations in astrocyte-conditioned medium (ACM) from healthy subject- and sporadic ALS-derived astrocytes were assessed. The levels of selected proteins of the canonical mTOR-autophagy pathway and autophagosome number were also evaluated. Then, we used pharmacological activation and inhibition of the mTOR-autophagy pathway in astrocytes derived from both control subjects and ALS patients to predict the role of autophagy in mediating aberrant astrocyte cytokine secretion. The present study confirmed that sporadic ALS patient iPSC-derived astrocytes demonstrated motor neuron toxicity. We also report that sporadic ALS patient iPSC-derived astrocytes exhibited increased secretion of the cytokines IL1*β*, TNF*α*, and IL6, which can result in motor neuron toxicity. Finally, an aberrant mTOR-autophagy pathway in sporadic ALS astrocytes mediated increased cytokine secretion by astrocytes. The findings of the present study suggested that an autophagy dysfunction in sporadic ALS astrocytes is one of the noncell autonomous mechanisms that may be able to mediate motor neuron toxicity in ALS pathogenesis.

## 2. Materials and Methods

### 2.1. iPSC Generation and Neuronal Differentiation

Venous blood was obtained from three ALS patients (HCHB-003, ALSHB-004, and ALSHB-001X) and three healthy control subjects (HCHB-002, HCHB-006X, and HCHB-005) who provided their informed consent for PBMC isolation. PBMCs (3 × 10^5^ cells) were transduced with the reprogramming factors OCT3/4, SOX2, c-MYC, and KLF4 using a CytoTuneTM-iPS 2.0 Sendai reprogramming kit (Thermo Fisher Scientific, USA) according to the manufacturer's instructions [25]. The trilineage differentiation potential and chromosomal aberrations in the PBMC-derived iPSCs were tested using a STEMdiff™ trilineage differentiation kit (StemCell Technologies) and by standard G-band karyotyping, respectively [26]. Karyotyping was performed by the Department of Clinical Human Genetic Center (Fourth Hospital of Shijiazhuang, China).

### 2.2. Neural Stem Cell (NSC), Astrocyte, and Motor Neuron Differentiation and Identification

For each subject, three iPSC clones were independently differentiated into NSCs. iPSCs were differentiated into neural stem stems (NSCs) as follows. Pluripotent stem cell (PSC) neural induction medium (Gibco) was used to induce iPSCs to transition to NSCs according to the manufacturer's instructions. Briefly, iPSCs were cultured in PSC neural induction medium on a Geltrex-coated 6-well plate, and the medium was replaced every other day. After 7 days, iPSCs differentiated into NSCs. The identity of NSCs was determined using IF staining for the markers nestin and SOX2 [SRY (sex-determining region Y)-box 2]. Subsequently, NSCs were cultured in NSC expansion medium (Table 1).

To differentiate NSCs into astrocytes, NSCs were plated on a Geltrex-coated 6 cm plate at a density of 1.25 × 10^6^ cells in NSC expansion medium. 24 h later, NSC expansion medium was replaced with astrocyte induction medium (AIM). The AIM treatment lasted 21 days, during which the medium was replaced every other day. When confluence was reached, the cells were replated in fresh Geltrex-coated flasks at a dilution of 1 : 4. At the end of this period, AIM was replaced with astrocyte medium. Astrocytes were then cultured for at least 5 weeks to obtain mature cells and used in the experiments. The identity of NSC-derived astrocytes was confirmed using IF staining for anti-glial fibrillary acid protein (anti-GFAP) and S100 calcium binding protein B (S100*β*). Astrocytes aged 45-55 days in vitro (DIV) were used for the experiment. An iPSC clone (HCHB-002) was randomly selected to differentiate into the motor neurons. iPSCs were differentiated first into the motor neuron progenitors (MNPs) and then from MNPs to the motor neurons as described by Du et al. [27]. The identity of the motor neurons was confirmed using IF staining for the marker choline acetyltransferase (ChAT).

### 2.3. Experimental Grouping

Upon reaching 90% confluence, each astrocyte group (healthy control subjects = 3 and ALS patients = 3) was passed into three subgroups as follows: rapamycin, 3-methyladenine (3-MA), or no autophagy modulator ATM (-). Each subgroup was analyzed in triplicate. Astrocytes were incubated in serum-free astrocyte medium for 1 h and then treated with rapamycin, 3-MA, or serum-free medium. The rapamycin groups were treated with 200 nM rapamycin for 48 h [28], and the 3-MA groups were treated with 5 mM 3-MA for 48 h [29]. Various treatments were applied depending on experimental objectives. Each experiment was repeated three times.

#### 2.3.1. Enzyme-Linked Immunosorbent Assay (ELISA)

Astrocytes from patient and control subjects were seeded at equal concentrations in a 10 cm culture dish. 24 h later, astrocytes were treated with rapamycin, 3-MA, or serum-free medium for 48 h. Cell culture media were collected and centrifuged at 500×*g* at 4 °C for 10 min. ACM was collected, and the concentrations of TNF*α*, IL1*β*, and IL6 were measured using human TNF-*α* (ABclonal Technology, cat. no. RK00030), human IL-1*β* (ABclonal Technology, RK00001), and human IL-6 (ABclonal Technology, RK00004) ELISA kits. Briefly, microtiter wells were precoated with an appropriate capture antibody and rinsed three times with wash buffer. One hundred microliters (100 *μ*L) of either test samples (culture medium supernatant) or various concentrations of the standard proteins were placed in the microtiter wells and incubated for 2 h. The wells were again washed three times and then incubated with a corresponding biotin-conjugated antibody for 1 h at 37 °C. Following three rinses with wash buffer, streptavidin-HRP solution was added to each well and incubated at 37 °C for 30 min. After three rinses with wash buffer, chromogenic 3,3′,5,5′-tetramethylbenzidine (TMB) solution was added to each well and incubated in the dark for 20 min at 37 °C. Stop solution was added to each well with substrate solution, and a TECAN microplate reader (model SPARK, A-5082, Austria) was used to determine the absorbance within 5 min at 450 nm; the correction wavelength was set at 570 nm. The concentrations of the cytokines in the test samples were then computed based on the linear plots of the absorbance versus concentrations of the standard.

#### 2.3.2. Neurosupportive Potential of Astrocyte ACM

The MTT (3-(4,5-dimethylthiazolyl-2)-2,5-diphenyltetrazolium bromide) assay was used to assess the effects of astrocyte-conditioned medium (ACM) and a cytokine medium preparation on the viability of the motor neurons and the effect of rapamycin and 3-MA on control astrocytes. Astrocytes from healthy control subjects and ALS patients were cultured in 6-well plates for 48 h in basic neural medium (Table 1). The medium used to culture astrocytes served as astrocyte-conditioned medium. To rule out a possible influence of the degradation of the medium components on the results, fresh medium (basic neural medium) and control medium (basic neural medium incubated for 48 h at 37 °C) were used as the controls. Cytokine medium was formulated as follows. Considering the concentrations of IL1*β*, IL6, and TNF*α* in ACM of astrocytes of ALS patients and healthy control subjects, IL1*β*, IL6, and TNF*α* were dissolved in motor neuron induction basic medium at the final concentrations of 58 pg/ml, 185 pg/ml, and 57 pg/ml, respectively. Motor neurons (2×10^3^ cells per well) were seeded in 96-well plates in triplicate. 24 h later, the motor neurons were treated with 200 *μ*l of ACM of ALS astrocytes, ACM of healthy control subject astrocytes, cytokine medium preparation, or motor neuron culture medium every other day for 8 days. Additionally, astrocytes from healthy control subjects (2×10^3^ per well) were seeded in triplicate in 96-well plates and cultured in basic neural medium. 24 h later, astrocytes were treated with astrocyte medium, astrocyte medium and vehicle, 200 nM rapamycin, or 5 mM 3-MA for 48 h. Afterwards, the MTT assay was performed as follows. Culture medium was aspirated, and 90 *μ*l of fresh culture medium followed by 10 *μ*l of 5 mg/ml MTT solution was added to each well. Motor neurons were incubated for 4 h. MTT-containing culture medium was aspirated, and 100 *μ*l of DMSO was added to each well to dissolve the formazan crystals. The culture plate was placed on a shaker for 10 min at a speed of 300 rev/min, and the absorbance was read at 490 nm wavelength using a TECAN microplate reader.

#### 2.3.3. Western Blotting

Patient and control subject astrocytes were seeded at equal concentrations in a 10 cm culture dish. 24 h later, astrocytes were treated with 200 nM rapamycin, 5 mM 3-MA, or serum-free medium for 48 h. Astrocyte cultures were lysed in ice-cold RIPA lysis buffer. Then, 20-30 *μ*g of protein was resolved by SDS–PAGE and transferred to the PVDF nitrocellulose membranes. After blocking of the PVDF membranes with 5% milk dissolved in TBST for 2 h at RT, the PVDF membranes were probed with the following antibodies (Table 2): anti-p-mTOR (Ser2448), anti-mTOR, anti-Ulk1, anti-pUlk1 (S757), anti-p62, anti-LC3B, and anti-GAPDH. The primary antibody incubation was performed overnight at 4 °C. After washing three times with TBST, the PVDF membranes were incubated with goat anti-rabbit horseradish peroxidase-conjugated secondary IgG antibodies. The immunoreactive proteins were detected using an enhanced *chemiluminescence* (ECL) kit (Service, G1221), and the images were obtained using an Amersham imager 600 (Cytiva) BD imaging system. Using GAPDH as an internal reference, the expression levels of p-mTOR (Ser2448)/p-mTOR, p-ULK1 (S757)/ULK1, p-Beclin-1 (Ser15)/p-Beclin-1, p62, and LC3BII/LC3BI were quantified by densitometric scanning using ImageJ software (version 1.53f51).

#### 2.3.4. Transmission Electron Microscopy (TEM)

Astrocytes from healthy control subjects and ALS patients were seeded in a 6 cm dish and cultured with serum-free astrocyte medium for 48 h. Astrocytes were collected by scraping and then centrifuged at 300×*g* for 5 minutes. The supernatant was discarded, and the cell pellet was washed twice with PBS. The cells were fixed with 2.5% glutaraldehyde at 4 °C for 4 h, washed thrice with 0.067 M phosphate buffer for 10–15-min, and fixed in 1% osmium tetroxide at 4 °C for 1-2 h. After three 10-15-min washes with 0.067 M phosphate buffer, the cells were dehydrated in a gradient of acetone concentrations (50%, 70%, 80%, and 90% for 10-15 min each and 100% for 10-15 min twice). The cell pellet was soaked in a 1 : 1 mixture of acetone:embedding solution for 1 h at 37 °C and then in a 1 : 3 mixture of acetone:embedding solution at 37 °C for 5 h, 37 °C for 24 h, and 60 °C for 48 h. Ultrathin sections of the pellet were prepared using an ultrathin slicer (Leica UC7). The sections were stained with uranyl acetate for 30-45 minutes and then with lead citrate for 5-30 minutes. Photomicrographs of the autophagosomes were acquired using transmission electron microscopy (model: Hitachi H-7500; voltage: 80 kV).

### 2.4. Immunocytochemistry

iPSCs, iPSC-derived trilineage cells, motor neurons, NSCs, and astrocytes of patients and healthy control subjects were fixed in 4% PFA for 10–15 min at room temperature (RT), washed twice with DPBS (Invitrogen, Carlsbad, CA, USA), permeabilized with 0.3% Triton X-100 in PBS for 10 min, and then blocked with 5% donkey serum (Jackson, YZ-017-000-121) for 30 min. iPSCs were incubated with DPBS-diluted primary antibodies (Table 2): anti-OCT4, anti-SOX2, anti-NANOG, anti-SSEA4, and anti-TRA-1-60. iPSC-derived trilineage cells were incubated with anti-AFP, anti-SMA, or anti-TUJ antibodies (Table 2). NSCs were incubated with anti-Nestin and anti-SOX2 antibodies. Astrocytes were incubated with anti-GFAP, anti-S100*β* and anti-p62 antibodies (Table 2). Motor neurons were incubated with an anti-ChAT antibody. All primary antibody incubations were performed overnight at 4 °C. After washing of the cells three times with PBS, the samples were incubated for 1 h at room temperature with the corresponding secondary antibodies: Alexa Fluor 488 AffiniPure donkey anti-rabbit IgG (H + L) and Alexa Fluor 594 AffiniPure donkey anti-mouse IgG (H + L). The cell nuclei were stained with DAPI for 10 min at RT. After washing with DPBS, fluorescent images were captured by an Olympus BX-53 fluorescence microscope. Photomicrographs of seven different fields were captured. The fluorescence intensity of the captured IF images was determined using ImageJ software (version 1.53f51).

### 2.5. Statistical Analysis

The data for absorbance, autophagosome number, concentrations of the cytokines in culture medium, and fluorescent intensity were expressed as the mean ± standard deviation. GraphPad Prism version 9 (GraphPad Software, LLC) was used for statistical analysis. An independent *t* test was used to compare the means of two groups. One-way analysis of variance (ANOVA) followed by Tukey's multiple comparison test was used to compare the means between three or more groups. A *p* value less than 0.05 was considered significant.

## 3. Results

### 3.1. Sporadic Patient iPSCs Convert to Abnormal Astrocytes after the Same Differentiation Program as iPSCs Derived from Healthy Individuals

**Figure 1:**
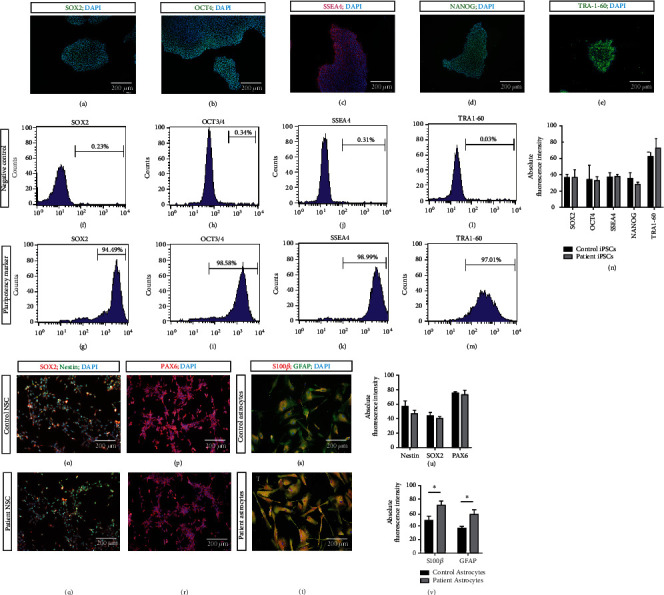
Identification of iPSC, NSC, and astrocyte. (a–e) Immunofluorescent (IF) staining of iPSC. SOX2 [SRY (sex-determining region Y)-box 2], OCT4 (octamer-binding transcription factor 4), SSEA4 ((stage-specific embryonic antigen 4), NANOG (Nanog homeobox), and TRA-1-60 (T-cell receptor alpha locus 60). (f–m) Flow cytometry of iPSCs for the markers (f, g) SOX2, (h, i) OCT3/4, (j, k) SSEA4, and (l, m) TRA-1-60. (n) Absolute fluorescent intensities of SOX2, OCT4, SSEA4, NANOG, and TRA-1-60 in the ALS patient and control iPSCs. (o–r) IF staining of patient and control NSCs for the markers (O&Q) SOX2, nestin, and DAPI. (p, r) PAX6 and DAPI. (s, t) IF staining of patient and control astrocytes for the markers (S) s100*β* (S100 Calcium Binding Protein B), GFAP (glial fibrillary acidic protein), and DAPI. (u) Absolute fluorescent intensities of Nestin, SOX2, and PAX6 in the ALS patient and control NSCs. (v) Absolute fluorescent intensities of s100*β* and GFAP in the AL patient and control astrocytes. ^∗^ represents *p* < 0.05.

To investigate the disease mechanisms and cellular pathology of ALS, we reprogrammed PBMCs of sporadic ALS patients (*n* = 3) and healthy individuals (*n* = 3) into iPSCs. Reprogrammed iPSCs of both ALS patients and control subjects showed an embryonic stem cell-like morphology. The two groups of iPSCs possessed the pluripotency potential evidenced by the positive staining of the pluripotent markers SOX2 [SRY (sex-determining region Y)-box 2], OCT4 (octamer-binding transcription factor 4), NANOG (Nanog homeobox), SSEA4 (stage-specific embryonic antigen 4), and TRA-1-60 (T-cell receptor alpha locus 60) (Figures 1(a)–1(m)). Patient and control subject iPSCs had comparable values of the mean absolute fluorescence intensity of the markers SOX2, OCT4, NANOG, SSEA4, and TRA-1-60 (Figure 1(n)). To generate astrocytes, iPSCs of both control subjects and patients were differentiated into neural stem cells (NSCs) after one week of neural induction. NSCs stained positive for the markers Nestin, SOX2, and PAX6 (paired box 6) (Figures 1(o)–1(r)). Patient and control subject NSCs had comparable values of the mean absolute fluorescence intensity of the markers Nestin, SOX2, and PAX6 (Figure 1(u)). Subsequently, NSCs were successfully differentiated into astrocytes with a uniformly fibroblastic morphology. According to the results of immunofluorescence staining, both patient and control subject iPSC-derived astrocytes expressed the corresponding markers, including GFAP and S100*β*, demonstrating the maturity and purity of astrocytes [30, 31]. All astrocytes immunoreacted with the marker antibodies. Interestingly, the mean fluorescence intensity of GFAP and S100*β* in astrocytes from patients was significantly higher than that in astrocytes from healthy control subjects (Figure 1(v)). GFAP and S100*β* can be used as biomarkers of astrocyte activation and proliferation in the central nervous system. This result suggested that the phenotype of patient astrocytes may differ from that of control subject astrocytes.

### 3.2. Patient iPSC-Derived Astrocyte-Conditioned Medium Is Toxic to Motor Neurons due to Astrocyte-Derived Cytokines

**Figure 2:**
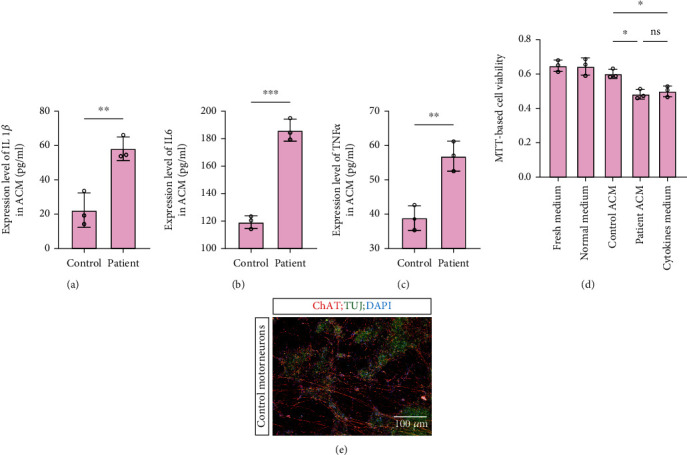
Cytokine concentration and neurosupportive potential of astrocyte-conditioned medium (ACM) from healthy control and sporadic ALS astrocytes. (a–) ELISA-determined concentrations, pg/ml, of (a) IL1*β* (b) IL6, and (c) TNF*α* of ACM. (d) MTT-determined viability of motor neurons treated with fresh medium, normal medium, control astrocyte ACM, patient astrocyte ACM, and cytokine medium. (e) IF staining of motor neurons for the markers ChAT (choline acetyltransferase), TUJ (*β*-tubulin III), and DAPI. ^∗^ represents *p* < 0.05, ^∗∗^ represents *p* value < 0.01, and ^∗∗∗^ represents *p* < 0.001. ELISA: enzyme-linked immunosorbent assay, MTT: 3-(4,5-dimethyl thiazolyl-2)-2,5-diphenyltetrazolium bromide.

Increasing focus of several noncell autonomous studies indicated that astrocytes may release certain factors that are directly toxic to the motor neurons. However, these factors remain largely unknown. To investigate whether astrocytes secrete proinflammatory factors, which are toxic to the motor neurons, we used ELISA to assess the concentrations of the cytokines (IL1*β*, IL6, and TNF*α*) in cell-conditioned medium of both patient and control subject astrocytes. We observed a significant increase in the levels of these cytokines secreted in patient ACM compared with that in control subject ACM (Figures 2(a)–2(c)).

To examine whether patient ACM has similar toxic effects on the motor neurons due to these cytokines, we differentiated control subject iPSCs into the motor neurons. Motor neurons showed positive staining for the markers ChAT and TUJ (Figure 2(e)). Astrocytes contribute to neuronal survival via numerous cellular processes. To assess the neurosupportive potential of astrocytes, the motor neurons were treated with conditioned medium from ALS astrocytes and control subject astrocytes for 48 h. Motor neurons were also treated with fresh and basic neural media by incubation at 37 °C for 48 h (control medium) or 0 h (fresh medium). Thereafter, an MTT assay was used to assess the optical density (OD) of dissolved formazan crystals, which corresponded to the viability of the motor neurons. Motor neurons treated with ACM of ALS astrocytes were characterized by a significantly lower OD than motor neurons treated with ACM of control subject astrocytes (Figure 2(d)). The OD values of the motor neurons treated with either normal medium or fresh medium were not different from the corresponding values obtained in the case of ACM of control subject astrocytes (Figure 2(d)). This result suggested that patient-derived astrocytes may secrete the factors that are toxic to the motor neurons. To further investigate whether astrocytes secrete proinflammatory factors to affect the motor neurons, we detected the toxic effect of reconstituted cytokine medium (which was formulated based on the cytokine concentrations in ACM of ALS astrocytes) on the motor neurons using the MTT assay. We observed that reconstituted cytokine medium had a comparable toxic effect on the motor neurons but a significantly worse effect than that of control subject ACM. Overall, the results suggested that increased cytokine concentrations in ACM of patient astrocytes may contribute to a reduction in the motor neuron viability.

### 3.3. The mTOR-Autophagy Pathway Is Altered in ALS Astrocytes

**Figure 3:**
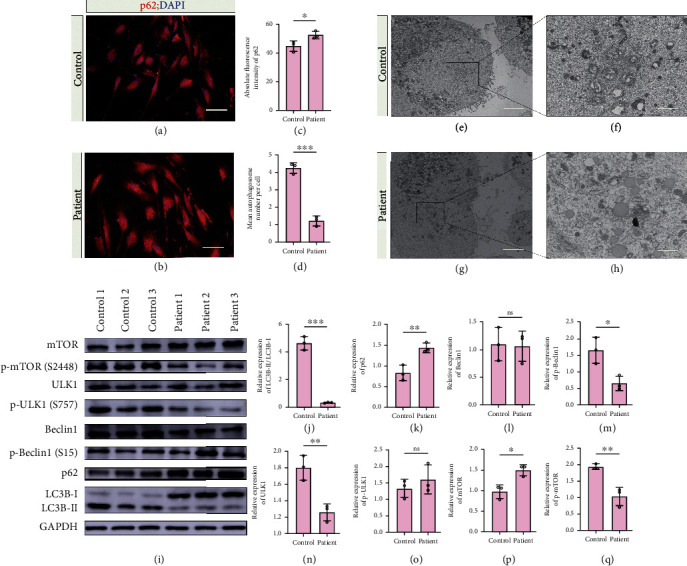
Autophagy-related variables in control and ALS astrocytes. (a) Immunoblots of LC3B-I/II and p62. (b,c) Bar graphs showing the densitometric quantification of (b) LC3B-II/I and (c) p62. (d, e) Immunofluorescent staining of p62 in (d) control and (e) ALS astrocytes. (f) Bar graph showing the absolute fluorescent intensities of p62 immunostaining in control and ALS astrocytes. (g–j) Transmission electron microscopy of autophagosomes in (g, h) control and (i, j) ALS astrocytes. (k) Bar graph showing the mean number of autophagosomes in control and ALS astrocytes. (l) Immunoblots of proteins in the mTOR-autophagy pathway: p-beclin1 (Ser15), beclin1, p-ULK1(Ser757), ULK1, p-mTOR (Ser2448), and mTOR. (m–r) Bar graphs showing the densitometric quantification (m) beclin1, (n) p-beclin1 Ser15, (o) ULK1, (p) p-ULK1 Ser757, (q) mTOR, and (r) p-mTOR (Ser2448). ^∗^ represents *p* < 0.05, ^∗∗^ represents *p* *value* < 0.01, and ^∗∗∗^ represents *p* < 0.001. mTOR: mammalian target of rapamycin; p-mTOR: phosphorylated mTOR; ULK1: Unc-51 like autophagy activating kinase 1; p-ULK1: phosphorylated ULK1; LC3B: microtubule-associated proteins 1A/1B light chain 3B; and GAPDH: glyceraldehyde 3-phosphate dehydrogenase.

Autophagy is a major regulator of cytokine secretion; however, precise mechanisms of the process in astrocytes remain unknown. The markers for the activation of canonical autophagy include the p62 and LC3B proteins. p62 is a multidomain signaling scaffold protein. In addition to several other functions, p62 binds to ubiquitinated substrates or autophagosome membranes via its UBA domain and LC3-binding domain, respectively. In the process of lysosomal degradation of autophagosomes during autophagy, substrate-bound p62 is degraded by proteolytic enzymes, causing the level of p62 to decrease. LC3B is a central protein in the autophagy pathway. During autophagy, LC3B-I is lipidated to form LC3B-II, which is also a marker of autophagosomes. LC3B-II level increases concomitant to an increase in the autophagosome numbers. Therefore, we aimed to explore whether autophagy is altered in patient astrocytes. Immunoblotting of the p62 and LC3B-II/LC3B-I proteins, immunofluorescence of the p62 protein, and/or TEM of autophagosomes were used to assess autophagy. The results obtained by western blotting of LC3B showed that the LC3B-II/LC3B-I ratio in ALS astrocytes was significantly lower than that in control subject astrocytes (Figures 3(i) and 3(j)), and the relative level of the p62 protein in ALS astrocytes was significantly higher than that in control subject astrocytes (Figures 3(i) and 3(k)). The IF results showed that the mean absolute fluorescent intensity of p62 in ALS astrocytes was higher than that in control subject astrocytes, which was consistent with these results (Figures 3(a)–3(c)). The results of TEM showed that the mean number of autophagosomes in ALS astrocytes was lower than that in control subject astrocytes (Figures 3(d)–3(h)). The results obtained by western blotting of LC3B showed that the LC3B-II/LC3B-I ratio in ALS astrocytes was significantly lower than that in control subject astrocytes (Figures 3(i) and 3(j)), and the relative level of the p62 protein in ALS astrocytes was significantly higher than that in control subject astrocytes (Figure 3(i) and 3(k)). These results suggested that autophagy was inhibited in ALS astrocytes.

In the canonical mTOR-autophagy pathway, the kinase mTOR phosphorylates and negatively regulates ULK1 (Unc-51 like autophagy activating kinase 1) at Ser757 to initiate autophagy. Dephosphorylated ULK1 phosphorylates and activates Beclin-1 at the Ser15 residue. Beclin-1 interacts with several proteins to activate the downstream autophagy pathway [32]. In the present study, the levels of the key proteins of the canonical mTOR-autophagy pathway, including Beclin-1, p-Beclin-1 (Ser15), ULK1, p-ULK1 (S757), mTOR, and p-mTOR (Ser2448), were assessed using western blotting. The levels of p-mTOR, ULK1, and p-Beclin-1 were significantly decreased, and the level of mTOR was significantly increased in patient astrocytes compared with those in control subject astrocytes; however, the levels of pULK1 and Beclin-1 were unchanged (Figures 3(i) and 3(l)–3(q)). These results suggested that in the mTOR-autophagy pathway, an increase in the mTOR levels coupled with a reduction in the ULK1 and p-Beclin-1 (Ser15) levels may correspond to a lower LC3B-II/LC3B-I ratio and to higher p62 levels in ALS astrocytes. It is possible that ULK1-dependent phosphorylation of Beclin-1 at the Ser15 residue, which is necessary for the activation of Beclin-1-dependent autophagy, is attenuated in ALS astrocytes.

### 3.4. Effect of Activation and Inhibition of Autophagy on Astrocyte-Related Cytokine Secretion

**Figure 4:**
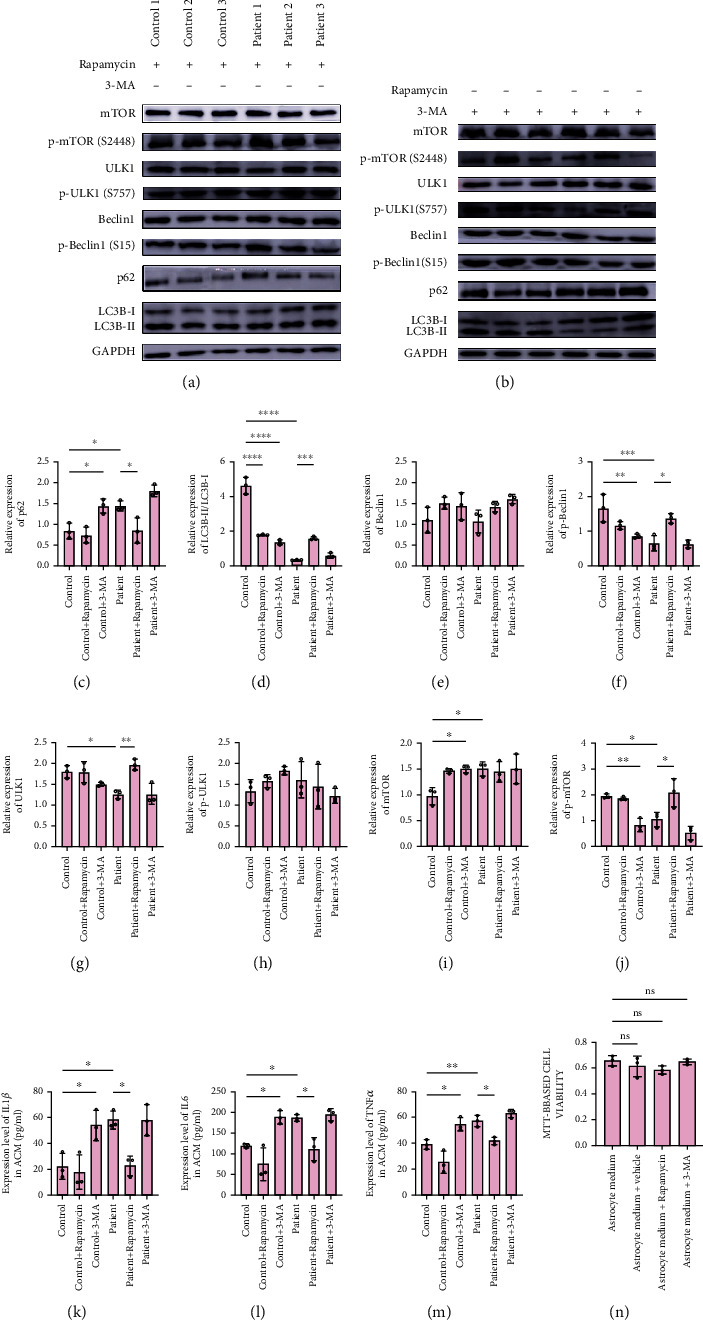
Effect of mTOR-autophagy pathway modulation in control and ALS astrocytes on cytokine concentration. (a, b) Immunoblots of proteins in the mTOR-autophagy pathway of sporadic ALS astrocytes and control astrocytes treated with (a) rapamycin and (b) 3-methyladenine. (c–j) Bar graphs showing the densitometric quantification of (c) LC3B-I/II, (d) p62, (e) beclin1, (f) p-beclin1 Ser15, (g) ULK1, (h) p-ULK1 Ser757, (i) mTOR, and (j) p-mTOR (Ser2448). (k–m) Astrocyte conditioned medium/ACM concentration of (k) interleukin 1*β*/IL1*β* (l) interleukin 6/IL6, and (m) tumor necrosis factor-alpha/TNF*α* of control and ALS astrocytes treated with or without 3-methyladenine/3-MA and rapamycin. (n) Viability of astrocytes treated with rapamycin or 3-MA. ^∗^ represents *p* < 0.05, ^∗∗^ represents *p* value < 0.01, and ^∗∗∗^ represents *p* < 0.001. mTOR: mammalian target of rapamycin; p-mTOR: phosphorylated mTOR; ULK1: Unc-51 like autophagy activating kinase 1; p-ULK1: phosphorylated ULK1; LC3B; microtubule-associated proteins 1A/1B light chain 3B; GAPDH: glyceraldehyde 3-phosphate dehydrogenase.

To test whether the involvement of an aberrant mTOR-autophagy pathway is related to aberrant cytokine secretion by ALS astrocytes, we attempted to assess cytokine secretion by control subject and patient astrocytes in vitro by pharmacologically modulating the mTOR-autophagy pathway in astrocytes using 3-methyladenine (3-MA) (a classical autophagy inhibitor) and rapamycin (a classical autophagy activator). First, using the MTT assay, we assessed whether rapamycin and 3-MA treatments are toxic to astrocytes. The mean OD values of dissolved formazan in vehicle-, rapamycin-, and 3-MA-treated astrocytes were not different from the OD values in the samples treated with fresh medium (Figure 4(n)).

Following rapamycin or 3-MA treatment, we assessed the levels of autophagy-related proteins in astrocytes using western blotting and the concentrations of the cytokines IL-1*β*, IL-6, and TNF-*α* in ACM using ELISA. 3-MA was shown to decrease the expression of p-mTOR and p-Beclin-1 and the LC3B-II/LC3B-I ratio and to increase the expression of mTOR and p62 in control subject astrocytes, indicating autophagy abnormalities (Figures 4(b)–4(d), 4(f), 4(i), and 4(j)). Unexpectedly, rapamycin had a similar effect on autophagy in control subject astrocytes, except causing a decrease in the LC3B-II/LC3B-I ratio (Figures 4(a), 4(c)–4(j)). It is possible that limited treatment with rapamycin was not sufficient to convert control subject astrocytes to ALS-like cells. However, rapamycin decreased the expression of p62 and increased the expression of p-mTOR, ULK1, and p-Beclin-1 and the LC3B-II/LC3B-I ratio in patient astrocytes; these values returned to the levels similar to those detected in control subject astrocytes, indicating the activation of autophagy (Figures 4(b)–4(d), 4(f), 4(i), 4(j)). Interestingly, 3-MA had no effect on autophagy in patient astrocytes (Figures 4(b)–4(j)). This phenomenon could have been caused by limited effects or other 3-MA-related mechanisms.

Subsequently, to investigate whether autophagy changes influence cytokine secretion, we detected IL1*β*, IL6, and TNF*α* in ACM after treatment with rapamycin or 3-MA. 3-MA increased the levels of IL1*β*, IL6, and TNF*α* in control subject ACM but not in patient ACM (Figures 4(k)–4(m)). Similarly, rapamycin decreased the levels of IL1*β*, IL6, and TNF*α* in patient ACM but not in control subject ACM (Figures 4(k)–4(m)). These results suggested that autophagy activation may be a suitable candidate to mitigate an increase in cytokine secretion in ALS astrocytes and that autophagy is a very important therapeutic target in ALS.

## 4. Discussion

Amyotrophic lateral sclerosis (ALS) leads to motor neuron-related pathologies, extra motor deficits and even death [2]. Noncell autonomous mechanisms involving non-neuronal cells, such as astrocytes, are involved in these processes. However, information about astrocyte-mediated motor neuron toxicity is biased because most of the studies about astrocyte-mediated motor neuron toxicity have revolved around familial ALS. Astrocyte-mediated motor neuron toxicity in sporadic ALS, which accounts for 90-95% of ALS patients, has been understudied. Clinical heterogeneity of ALS necessitates the investigations of the variations of the pathogenesis of both forms of ALS, which are known to facilitate the selection and design of therapeutics for the management of the two ALS forms. The present study demonstrated that sporadic ALS astrocytes exhibited elevated secretion of the cytokines IL1*β*, TNF*α*, and IL6, which may be involved in motor neuron toxicity. In addition, increased secretion of the cytokines in sporadic ALS astrocytes may be caused by an impaired mTOR-autophagy pathway. Inhibition of the mTOR-autophagy pathway in the control astrocytes mimicked cytokine secretion and most of the anomalies of the autophagy pathway in sporadic ALS astrocytes. Finally, the activation of the mTOR-autophagy pathway in patient astrocytes reverted the elevation of cytokine secretion and most of the anomalies of the mTOR-autophagy pathway compared to those detected in healthy control subject astrocytes.

Inflammation is characteristic for neurodegenerative diseases, including ALS, and the process can be mediated by glial cells, including astrocytes. Astrocytes may contribute to inflammation by serving as an inducible source of inflammatory cytokines, such as IL1*β*, TNF*α*, and IL6; these cytokines have been demonstrated to be involved in neurodegenerative diseases, including ALS pathogenesis [11, 12, 14]. The present study demonstrated that sporadic ALS astrocytes exhibited increased secretion of inflammatory cytokines TNF*α*, IL1*β*, and IL6. A cytokine cocktail medium was generated based on the concentrations of TNF*α*, IL1*β*, and IL6 detected in ALS astrocyte ACM and was shown to reduce motor neuron viability; this influence was comparable to the effects of conditioned medium of ALS patient astrocytes. Thus, increased cytokine secretion may be a noncell autonomous mechanism by which sporadic ALS causes motor neuron death. ALS astrocytes, particularly familial ALS astrocytes, have been shown to cause motor neuron toxicity. Cytokine-mediated motor neuron toxicity has also been demonstrated in familial ALS in the case of FUS-mutated astrocytes that secrete high levels of TNF*α* to cause MN toxicity [9]. Other noncell autonomous mechanisms have been demonstrated in familial ALS astrocytes. In C9orf72 ALS, astrocytes release extracellular vesicle-derived microRNAs to cause motor neuron toxicity [10]. Additionally, C9Orf72 astrocytes exhibit increased oxidative stress and senescence and secrete soluble factors to induce oxidative stress in wild-type motor neurons [7]. Astrocytes with an SOD1 mutation (hSOD1^G93A^) are selectively toxic to the motor neurons, and this toxicity involves prostaglandin D2 [33]. In the case of sporadic ALS, currently available references suggest that the content of extracellular vesicles, which are enriched in the SOD1, phospho-TDP-43, and FUS proteins, is increased in the sera of sporadic ALS patients [34]. The source of these proteins has not been investigated. Considering that both sporadic and familial ALS astrocytes, irrespective of the source, are toxic to the motor neurons, sporadic ALS astrocyte-mediated motor neuron toxicity may involve a mechanism similar to that reported in the case of familial ALS astrocytes. However, these mechanisms were not explored in the present study and may be explored in the future studies related to sporadic ALS astrocytes.

The etiology of astrocyte-mediated inflammation in neurodegenerative diseases is not well understood. It has been proposed that autophagy dysfunction may represent the etiology of astrocyte-mediated inflammation. Most of available references reported the potential of cytokines, such as TNF*α* [35, 36] and IL6 [37, 38], to modulate the mTOR-autophagy pathway. Reports about the modulatory effects of autophagy on the cytokines are scarce. Usually, the cells activate autophagy to break down defunct cell parts and cytoplasmic inclusions. Similar to other neurodegenerative diseases, ALS is characterized by the accumulation of cytoplasmic inclusions and misfolded proteins in both motor neurons and glia. Cell-specific impairment of autophagy was suggested to underlie the ALS pathogenesis. The present study demonstrated that sporadic ALS astrocytes exhibited impaired autophagy, which was evidenced by a reduction in the LC3B-II levels and LC3B-II/LC3B-I ratio and an increase in the p62 levels. The LC3B-II levels correlate with the number of autophagosomes, and the number of autophagosomes in sporadic ALS was reduced as expected. Impaired autophagy in sporadic ALS astrocytes reported in the present study was in agreement with the findings of Granatiero et al. [8] who reported that autophagy is impaired in SOD1^G93A^ ALS astrocytes [8]. Similarly, the LC3B-II levels were reduced in SOD1^G93A^ ALS astrocytes. The authors reported that p-ULK1 (S757) and p-mTOR (Ser2448) levels were increased, indicating the mTOR overactivation. In contrast, we demonstrated that the p-ULK1 (S757) levels did not change and that the p-mTOR (Ser2448) levels were reduced in sporadic ALS astrocytes. In sporadic ALS astrocytes in the present study, the phosphorylation of Beclin-1 at the serine 15 residue and the ULK1 levels were reduced, which could have induced the inactivation of the mTOR/ULK1/Beclin-1/p62/LC3B autophagy pathway. We suggest that the canonical mTOR-autophagy pathway is impaired in both sporadic and familial ALS astrocytes; however, the causes of the impairment of the pathway may be heterogeneous, stemming from increased or decreased mTOR activation and reduced phosphorylation of Beclin at Ser15. Noncanonical pathways, such as ULK1-independent and Beclin-1-independent autophagy pathways, have been demonstrated to influence LC3B. Future studies may assess these noncanonical autophagy pathways to elucidate astrocyte autophagy-mediated noncell autonomous toxicity in ALS pathogenesis.

## 5. Conclusion

The patient and control subject iPSC-derived astrocytes demonstrated their utility in mechanistic studies of ALS. Sporadic patient iPSC-derived astrocytes exhibited increased secretion of the cytokines IL1*β*, TNF*α*, and IL6, which demonstrated a neurotoxic potential. Aberrant secretion of the cytokines may be attributed to impaired autophagy due to an alteration in the mTOR/ULK1/Beclin-1 autophagy pathway, such as impaired phosphorylation of Beclin-1 at Ser15. The modulation of the pathway using rapamycin, but not 3-MA, significantly reduced cytokine secretion by patient iPSC-derived astrocytes. In addition, the results of the present study suggested that rapamycin can be considered a repurposed candidate drug for the management of inflammation-induced neuron-related pathologies in ALS pathogenesis.

## Figures and Tables

**Table 1:** Culture medium and components.

Medium	Components
Basic neural medium	Neurobasal medium (Gibco) supplemented with 10 mM CHIR99021, 10 mM DMH-1, 10 mM SB431542, 10 mM RA, 5 mM purmorphamine, and 500 mM valproic acid (VPA)
Astrocyte induction medium	DMEM/F12 (11330-032, Thermo Fisher Scientific, MA, USA), N2 supplement (17502001, Thermo Fisher Scientific, MA, USA) and 10% fetal bovine serum (FBS) (12657029, Thermo Fisher Scientific, MA, USA)
Astrocyte maintenance medium	DMEM/F-12 and FBS (9 : 1)
PSC neural induction medium	Neurobasal medium (Gibco) supplemented with 10% neural induction supplement (Gibco)]
NSC maintenance medium	Neurobasal medium, DMEM/F-12, N2, and B27 (48.5 : 48.5 : 1 : 2)

**Table 2:** The company, Catalogue number and RRID of antibodies.

Antibody	Host	Dilution	Company, catalogue #, and RRID
Alexa Fluor 488 AffiniPure Donkey Anti-Rabbit IgG	Donkey	1 : 200	Jackson ImmunoResearch Laboratories, Inc. 711-545-152, RRID AB_2313584
Alexa Fluor 594 AffiniPure Donkey Anti-Mouse IgG (H + L)	Donkey	1 : 200	Jackson ImmunoResearch Laboratories, Inc., 715-585-150, RRID AB_2340854
Anti-AFP	Rabbit	1 : 200	GeneTex GTX72748, RRID_374821
Anti-beta (*β*)-actin	Mouse	1 : 1000	Cell signaling technology (CST), cat# 4967
Anti-C3	Rabbit	1 : 100	ABclonal A13283, RRID AB_2760135
Anti-GFAP	Mouse	1 : 200	BD Pharmingen cat# 556327, RRID AB_396365
Anti-LC3B	Rabbit	1 : 1000	Gene Tex, cat# GTX127375
Anti- mTOR (7C10)	Rabbit	1 : 1000	Cell signalling technology (CST), cat# 2983
Anti-NANOG	Rabbit	1 : 200	Abcam ab21624, RRID: AB_446437
Anti-nestin	Mouse	1 : 200	Gene Tex cat# GT935, AB_2716635
Anti-OCT4	Rabbit	1 : 200	Abcam cat# ab18976, RRID: AB_444714
anti-phospho-mTOR (Ser 2448)	Rabbit	1 : 1000	Cell signaling technology (CST), cat# 2971
Anti-SOX2	Rabbit	1 : 200	Abcam ab97959, RRID: AB_2341193
Anti-SMA	Mouse	1 : 200	Dako M0851, RRID AB_2223500
Anti-S100*β*	Rabbit	1 : 100	Gene Tex, GTX129573, RRID AB_2886037
Anti TRA-1-60	Mouse	1 : 200	Abcam ab16288, RRID: AB_778563
Anti-TUJ	Rabbit	1 : 200	Abcam, T2200, RRID: AB_10704920
Anti-Ulk1	Rabbit	1 : 1000	GeneTex, cat # GTX16974
Peroxidase-conjugated AffiniPure Goat Anti-Mouse IgG (H + L)	Goat	1 : 10000	Jackson ImmunoResearch Laboratories, Inc. 115-035-003, RRID: AB_10015289
Peroxidase-conjugated AffiniPure Goat Anti-rabbit IgG (H + L)	Goat	1 : 10000	Jackson ImmunoResearch Laboratories, Inc. 111-035-144, RRID: AB_2307391
DAPI		1 : 1000	Sigma, D9542

## Data Availability

The data used to support the findings of this study are available from the corresponding author upon request.
